# Controlling HIV-1: Non-Coding RNA Gene Therapy Approaches to a Functional Cure

**DOI:** 10.3389/fimmu.2015.00474

**Published:** 2015-09-16

**Authors:** Chantelle L. Ahlenstiel, Kazuo Suzuki, Katherine Marks, Geoff P. Symonds, Anthony D. Kelleher

**Affiliations:** ^1^The Kirby Institute, UNSW Australia, Sydney, NSW, Australia; ^2^Immunovirology Laboratory, St. Vincent’s Centre for Applied Medical Research, Darlinghurst, NSW, Australia; ^3^Calimmune Inc., Darlinghurst, NSW, Australia

**Keywords:** HIV-1 transcription, latency, non-coding RNA, siRNA, shRNA, humanized mouse model, gene therapy, clinical trials

## Abstract

The current treatment strategy for HIV-1 involves prolonged and intensive combined antiretroviral therapy (cART), which successfully suppresses plasma viremia. It has transformed HIV-1 infection into a chronic disease. However, despite the success of cART, a latent form of HIV-1 infection persists as integrated provirus in resting memory CD4^+^ T cells. Virus can reactivate from this reservoir upon cessation of treatment, and hence HIV requires lifelong therapy. The reservoir represents a major barrier to eradication. Understanding molecular mechanisms regulating HIV-1 transcription and latency are crucial to develop alternate treatment strategies, which impact upon the reservoir and provide a path toward a “functional cure” in which there is no detectable viremia in the absence of cART. Numerous reports have suggested ncRNAs are involved in regulating viral transcription and latency. This review will discuss the latest developments in ncRNAs, specifically short interfering (si)RNA and short hairpin (sh)RNA, targeting molecular mechanisms of HIV-1 transcription, which may represent potential future therapeutics. It will also briefly address animal models for testing potential therapeutics and current gene therapy clinical trials.

## Introduction

Non-coding RNAs (ncRNAs) are widely accepted as important regulators of cellular processes acting through post-transcriptional control of protein expression. The coding regions translated into protein of the human genome account for ~2%, while over 90% of the non-coding genome is reported to be utilized for transcription (1–4). Consequently, ncRNAs make up the majority of the mammalian transcriptome (2) and are reported to function in the transcriptional regulation of gene expression during embryogenesis, cell differentiation (5) and in response to external stimuli, particularly virus infection (6). NcRNAs are classified as regulatory or infrastructural (ribosomal and transfer RNAs). The regulatory class can be divided into small ncRNAs (<200 nucleotides) and long ncRNAs (>200 nucleotides) (7), with small ncRNAs being further categorized into microRNA (miRNA), short interfering RNAs (siRNAs), and antisense RNAs (asRNAs). This review will primarily focus on siRNAs and short hairpin RNAs (shRNAs) as there have been several recent reviews of the roles of long ncRNAs and other small ncRNAs (8, 9).

The HIV-1 5′ long terminal repeat (LTR), which acts as the promoter for integrated virus, consists of ~450 base pairs. It includes multiple binding sites for host transcription factors, including NF-κB, NFAT, SP1, and AP-1, which act to enhance viral transcription. HIV-1 transcription from integrated provirus is a highly controlled process, regulated by a number of epigenetic modifications, with recent studies identifying the involvement of ncRNAs. The major HIV-1 transactivation protein, Tat, dramatically enhances viral transcription by binding to a dynamic stem loop structure in viral RNA, the transactivation response element (TAR), which is also coded within the 5′LTR. In contrast, in cells harboring latent HIV-1, transcription is severely restricted and the 5′LTR promoter region carries a specific epigenetic profile, which includes increased histone methylation, decreased histone acetylation, which follows the recruitment of histone deacetylases (HDACs). These biochemical changes involve the histones of two nucleosomes (nuc0 and nuc1) that associate with specific regions of the 5′LTR. In particular, these changes are associated with repositioning of nuc1 (10–15). In latent virus, nuc1 overlies the transcription start site, whereas in actively transcribing virus, the demethylated/acetylated nucleosome slides upstream of the transcription start site (13).

This review aims to highlight a highly innovative approach to current HIV-1 therapies that utilizes small RNAs, which may represent an alternative strategy to eradication through a functional cure. We will discuss the latest developments in ncRNAs, specifically siRNA and shRNA, for targeting the molecular mechanisms of HIV-1 transcription as well as briefly address humanized murine models, which provide a vehicle to assess potential therapeutics, current gene therapy clinical trials, and future directions of promising therapeutics.

## Limitations of HIV-1 Therapy

Combined antiretroviral therapy (cART) has revolutionized the treatment of HIV-1 infection in the developed world, changing a fatally acute disease into a manageable chronic condition. However, there are significant caveats accompanying lifelong cART therapy, which is currently necessary to control HIV-1 replication. These include the ongoing burden of compliance, drug toxicities, and residual excess morbidity and mortality, mostly due to serious non-AIDS events (16). Whilst effective cART rapidly suppresses the plasma viral load (pVL) to near undetectable levels (17) and permits reconstitution of immune cells (18), the integrated HIV-1 provirus DNA remains essentially unaffected, and therefore provides a viral reservoir, from which recrudescence can occur upon interruption of cART (19, 20). The current barrier to HIV eradication is this reservoir that persists in resting memory CD4^+^ T cells and cells of myeloid lineage (17, 21–24). While multiple approaches have been explored to surmount this obstacle, including early intervention with cART (25), cART intensification (18, 26–30), and purging of the reservoir using reactivation strategies (31–35), the fundamental limitation remains that in the overwhelming majority of patients, the virus rebounds with cessation of cART (36).

## Current Therapeutic Approaches to Achieve a Functional Cure

While the ultimate goal is often stated to be the eradication of HIV, that is achieving a sterilizing cure, recent studies have suggested a more realistic approach might be to achieve a functional cure, whereby therapeutic interventions mediate a clinical state of undetectable pVL in the absence of cART. So far, the “Kick and Kill Approach” to eradication is the most studied to date, with the objective of reactivating the latent reservoir while the patient continues cART. The expected outcome is that infected cells would be killed either directly by virus reactivation or through the cytotoxic T-lymphocyte immune response as viral proteins are expressed on the cell surface following reactivation. The presence of cART should prevent new cells becoming infected. The desired result is reduction in or elimination of the latent reservoir. However, despite various interventions including pan T cell activation through OKT3 (37), recombinant IL-2 (38), or IL-7 (34, 39), and activation of the protein kinase C (PKC) or protein kinase B pathways using prostatin, bryostatin, or disulfram, respectively (40), these approaches have had only limited or no success in achieving a reduction in the size of the latent reservoir.

Most “kick and kill” approaches have focused on driving virus reactivation by modifying the epigenetic profile of the virus in latent reservoir by using HDAC inhibitors (HDACi). These have included valporic acid (VPA), suberoylanilide hydroxamic acid (SAHA, vorinostat), romidepsin, and panobinostat (41–46). *In vitro* studies have shown substantial reactivation of integrated virus in cells from certain patients. However, *in vivo* the extent of viral reactivation has been limited and these drugs induce substantial off-target effects with significant non-specific host gene activation (47, 48). This has been most clearly demonstrated for SAHA, which was observed to inhibit CTL function (49), and would therefore impede CTL mediated “kill” of previously latently infected cells following the HDACi-induced “kick” (49). These findings all point to the need to dissect more precisely the molecular mechanisms involved in HIV-1 latency, particularly reactivation, to develop a more specific and targeted approach in manipulating the viral reservoir.

## Post-Transcriptional Gene Silencing of HIV-1 by si/shRNAs

A major limitation of the post-transcriptional gene silencing (PTGS) approach is the opportunity for viral escape due to targeting at the mRNA level, which allows the transcription process to potentially incorporate resistance mutations in the targeted sequence. To address this limitation, a similar strategy to combat HIV-1 drug resistance has been adopted, using dual or triple combination therapy of anti-HIV-1 shRNAs and/or other anti-HIV-1 gene therapeutics (50–52).

The first Phase 2 cell-modified gene therapy clinical trial using a combination approach involved a *tat*-*vpr*-specific anti-HIV ribozyme, termed OZ1, delivered in autologous CD34^+^ HPSCs (53). Although there were no significant viral load differences reported between the OZ1 and placebo groups, this study demonstrated cell-modified gene therapy was safe and biologically active, with no adverse events and higher CD4^+^ cell counts in the OZ1 group (53). A substantially decrease in the blood therapeutic gene level throughout the 100-week trial (53) may provide an explanation for the lack of effect.

A combinatorial approach uses three anti-HIV-1 shRNAs, each specifically targeting highly conserved regions of the HIV Integrase, Protease and *tat-rev* genes, delivered in a single LV construct termed R3A (52). This study first demonstrated multiple shRNAs being efficiently expressed in a single LV construct, if expression is driven by different promoters for each shRNA, e.g., the human H1, 7SK, or U6 polymerase III promoters and the human U1 polymerase II promoter each driving one of four shRNAs (52). Potent inhibition of HIV-1 was reported *in vitro*, with combined LV shRNAs constructs showing greater inhibition compared to single LV shRNA constructs (52). More recently, a preclinical *in vivo* study demonstrated safety of the R3A, triple shRNA expressing construct, using a Balb/c Rag2(−/−) IL-2Rγc(−/−) (BRG) humanized mouse model (54). Future clinical trials are planned to develop this potential therapeutic.

Another combinatorial HIV-1 approach involves shRNA targeting the *ccr5* gene and the C46 peptide fusion inhibitor in a LV construct termed LV sh5/C46 (or Cal-1), which has been developed by Calimmune Inc. It will be discussed further in the humanized murine model section.

## Transcriptional Gene Silencing of HIV-1 by si/shRNAs

The transcriptional gene silencing (TGS) approach has several distinct advantages over a PTGS approach; first, it directly targets the integrated virus, locking transcription, thus the opportunity for virus escape is minimized; second, epigenetic changes induced during TGS are heritable resulting in daughter cells maintaining the suppressive phenotype, providing a prolonged therapeutic response; and third, the exquisite sequence specificity of promoter-targeted si/shRNAs reduces the chance of non-specific off-target effects and toxicities.

Following the first identification of small interfering RNA-induced TGS in tobacco plants just over 10 years ago (55), the field has rapidly developed with multiple studies now identifying the phenomena in other plant species (*Arabidopsis*) (56, 57), fission yeast (*Schizosaccharomyces pombe*) (58), flies (*Drosophila*) (59), and nematode worms (*Caenorhabditis elegans*). Most recently, we and others have demonstrated TGS also occurs in human cells (60–66). We were the first group to report TGS could be induced in active HIV-1 infection through siRNA targeting the tandem NF-κB binding motifs in the 5′LTR viral promoter region (67). Subsequently, several studies have also identified targets for siRNAs that induce TGS in the HIV-1 promoter, specifically LTR-247, LTR-362, LTR-366 (68), and S4-siRNA (69). Of note, promoter-targeted siRNAs LTR-366 and S4-siRNA also target sequences in the NF-κB binding motif.

Our extensive investigations have demonstrated TGS of HIV-1 using the promoter-targeted si/shRNA PromA sequence complementary to the 5′LTR tandem NF-κB motifs initially *in vitro* using cell lines (64, 67, 70–72) and more recently in an *in vivo* humanized murine model (73). Characterization of the effects of si/shPromA revealed prolonged and profound TGS with up to a 1000-fold decrease in viral replication after a single siRNA transfection or retroviral-delivered shRNA construct. We also reported si/shPromA suppressed viral mRNA expression and *de novo* virion formation, while proviral DNA was still detected, indicating that virus suppression occurs post-integration. In further studies, we confirmed si/shPromA-mediated suppression of HIV-1 occurred via the TGS pathway using nuclear run-on assays to confirm transcription initiation rates, which clearly distinguished transcriptional suppression in si/shPromA treated HIV-1 infected nuclei from potential post-transcriptional effects (67, 72). Similarly, our investigations using a 3′LTR HIV-1-driven luciferase reporter construct also confirmed a limited contribution of PTGS to the observed virus suppression (72). Finally, chromatin immunoprecipitation (ChIP) analyses provided insight into the mechanism underlying the TGS, demonstrating that silencing was associated with increased histone methylation (H3K9me2 and H3K27me3), decreased histone acetylation, and recruitment of HDAC1 in the 5′LTR promoter region (64, 67, 72, 74). These structural histone changes are consistent with epigenetic-induced latent HIV infection (10–15, 72).

A recent study by Singh et al. using S4-siRNA targeted to the unique triple repeat of NF-κB binding motifs found in subtype C virus demonstrated long-term suppression of HIV-1 (69). The mechanism of silencing was shown *in vitro* to act through TGS, as determined by ChIP analysis of histone methylation, which revealed enrichment of H3K9me2 and H3K27me3 in S4-siRNA-transfected reporter cell lines (containing the subtype C LTR, luciferase reporter, and subtype C Tat protein expression cassette), but no enrichment in mock- or control siRNA-transfected cells (69). The suppressive effect was confirmed *ex vivo* in human PBMCs using four different subtype C isolates, which were shown to produce significantly lower p24 levels when transfected with S4-siRNA compared to mock- or control siRNAs (69). Interestingly, while a single mismatch at position 15 of the S4-siRNA sequence and a double mismatch at positions 1 and 15 were sufficient to disrupt the suppressive effect, one particular single mismatch at position 1 of the target sequence showed no disruption of the suppressive effect (69). This is intriguing considering the highly sequence specific nature of siRNAs and our experience of single mismatches disrupting any prolonged suppressive effect with loss of responses by ~6 days (75). Nevertheless, the unique S4-siRNA presents an interesting potential therapeutic approach in the specific targeting of subtype C, which is present in ~50% of the world’s HIV-positive population and demonstrates that much is still to be learnt regarding the properties of a targeted sequence that allows effective induction of viral silencing by promoter-targeted siRNAs.

We have also recently discovered another novel highly conserved sequence in the 5′LTR promoter region (including subtype C), upstream of siPromA, which when targeted by an si/sh RNA dubbed 143, induces potent transcriptional suppression of HIV associated with epigenetic modifications similar to those induced by siPromA (75). ChIP analyses revealed that the epigenetic changes induced by siRNA143 consisted of increased levels of both H3K27me3 and H3K9me3, reduction in H3K9Ac, and recruitment of Argonaute-1 (75), which are all characteristic heterochromatin marks observed during shRNA-induced TGS. Further, these changes in histone methylation and acetylation are consistent with epigenetic modifications found in latent HIV-1 infection. Following both siRNA PromA and 143 transfections, we observed that silencing can be partially reversed by highly potent, but also highly toxic, HDACi, such as trichostatin A (TSA), but not by those undergoing clinical trial evaluation, such as vironostat (SAHA) (75). Further, TNF at supra-physiological concentrations partially reduced the silencing. Additionally, using the J-Lat 9.2 cell latency model transduced with shPromA and/or sh143, we also observed robust resistance to viral reactivation by various stimuli, including SAHA and/or TNF, used at pharmacological or physiological concentrations (75). These observations are important in the context of the proposed gene-therapy applications as successful therapy will require sustained viral silencing despite activation of CD4^+^ T cells by inflammatory, homeostatic, or immune response proteins.

Identification of a second effective TGS target provides the opportunity to combine the shRNA targets (shPromA and sh143) in a single therapeutic. This combinatorial approach addresses concerns of any sequence-based target by covering HIV sequence variability. The PromA and si143 sequence targets are both highly conserved, however where variability does occur, the two constructs are complementary; e.g., subtype C viruses have a single nucleotide deletion at position 14 of the PromA target, while the sh143 sequence target has minimal subtype C variability. Thus, a multiplexed approach, delivered by a single LV construct expressing multiple shRNAs simultaneously, would likely provide increased viral coverage and more entrenched enforcement of epigenetic changes, which more robustly resist viral reactivation from alterations in the host’s inflammatory or immunological status. We are currently testing these constructs in humanized mouse models, described below.

## Humanized Mouse Models for Assessing Potential HIV-1 Therapeutics

**Figure 1:**
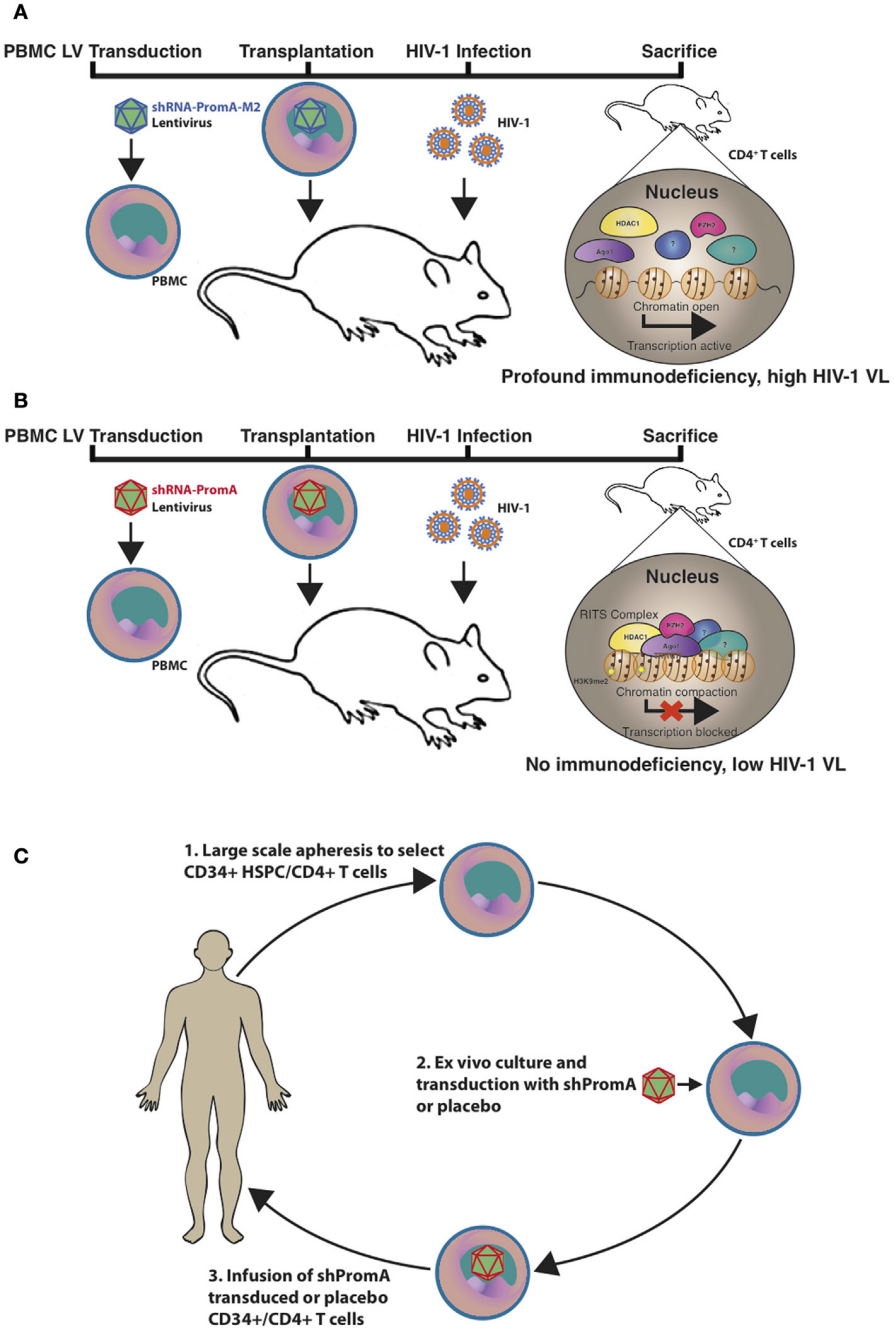
**Schematic representation of the *in vivo* effects of a promoter-targeted siRNA approach in a humanized mouse model and envisaged gene therapy approach**. Replication-incompetent lentivirus carrying **(A)** the inactive control shPromA-M2 or **(B)** active shPromA is transduced into healthy control human CD4^+^ T cells. Transduced CD4^+^ T cells are transplanted into (NOD)/SCID/Janus kinase 3 (NOJ) knockout mice and engraftment ensues. The humanized mice are then challenged with HIV-1 and sacrificed 14 days post challenge. The shPromA antisense strand (red), associates with Ago1 (purple) and other RITS-like complex components (HDAC – yellow and EZH2 – pink) and induces heterochromatin formation with methylation marks (H3K9me2, indicated by stars) in the targeted HIV-1 promoter region. This process suppresses HIV-1 transcription and results in protection of CD4^+^ T cells, which results in lower pVL in mice transplanted with shPromA compared to control shPromA-M2 lentivirus-transduced PBMCs. **(C)** Our envisaged gene therapy approach with the future shPromA and/or sh143 TGS-inducing targets involves initial apheresis to obtain and select CD34^+^ HSPC and/or CD4^+^ T cells, which are then cultured *ex vivo* and transduced with the multiplexed shRNAs. The transduced cells are then infused back into the patient, whereby HIV-1 will be locked down in a latent-like state.

Animal models for assessing HIV-1 therapeutics include various humanized murine models and non-human primates. Although the latter species contain host-restriction factors that impede HIV-1 replication and experiments performed using this model must instead use the closely related Simian immunodeficiency virus or chimeric Simian/HIV (SHIV) (76). We recently utilized a (NOD)/SCID/Janus kinase 3 (NOJ) knockout humanized mouse model to demonstrate *in vivo* TGS activity of shPromA, delivered via a LV (Figure 1) (73). NOJ knockout mice were reconstituted with human PBMCs transduced with the shPromA carrying lentiviral construct, which was processed into mature siPromA by cellular ribonucleases (77). HIV-1_JRFL_ challenge of mice reconstituted with the PromA-M2 inactive control transduced PBMCs showed acute HIV-1 infection (Figure 1A) as determined by high pVL, CD4^+^ T cell depletion and extensive immunodeficiency (78, 79). In stark contrast, mice reconstituted with shPromA-transduced PBMCs demonstrated significantly lower pVL and normal human CD4^+^ to CD8^+^ T cell ratios in mononuclear cells recovered from the peritoneal cavity and spleen at sacrifice 14 days post HIV-1 challenge (Figure 1B) (73). This corresponds to a protective effect in the form of an induced HIV-1 “latent-like” state, which locks down active virus transcription even in this acute model of HIV-1 infection.

Our aim is to generate sufficient data using the humanized mouse model to merit further therapeutic development of these TGS-inducing constructs. An alternative humanized murine model, which circumvents the highly acute infection reported in NOJ knockout mice, is the humanized BLT murine model. This model uses the non-obese diabetic (NOD)/SCID, common gamma chain −/− (NSG) mouse, humanized with implanted bone marrow, fetal liver, and thymus tissue (BLT) and results in systemic repopulation with human T cells, B cells, monocytes/macrophages, and dendritic cells (76, 80). This system has been used to perform *in vivo* studies of autologous CD34^+^ HSPCs transduced with the H1-CCR5 shRNA 1005 vector and showed effective down-regulation of the HIV-1 co-receptor, CCR5, which protected mouse-derived human splenocytes *ex vivo* (80) and CD4^+^ T cells *in vivo* (81) from CCR5-tropic HIV-1 infection. This CCR5-shRNA vector has also been analyzed in non-human primates by delivery through HSPC transplantation (82). Although this required a single nucleotide mutation in the human CCR5 shRNA 1005 sequence to match the rhesus macaque CCR5 target sequence, this analysis successfully showed specific inhibition of rhesus macaque CCR5 expression, with no modulation of human CCR5 expression observed (82).

The H1-CCR5 shRNA 1005 vector has been further developed to include an additional anti-HIV target in the form of a sequence that encodes for the C46 fusion inhibitor. This HIV-1 entry inhibitor is a mimetic derived from the peptide sequence of the C-terminal heptad repeat of HIV-1 gp41, which interacts with the N-terminal coiled-coil domain of the intermediate HIV-1 gp41 to block the six-helix bundle formation and subsequent fusion between HIV-1 envelope and the host cell membrane. C46 has been tested in Phase 1 clinical trials without any adverse effects in HIV-1 positive patients infused with autologous T cells transduced with C46 expressing retrovirus vector (83). Combination of H1-CCR5 shRNA 1005 and C46 into a single LV termed LVsh5/C46 (or Cal-1) has been tested in a preclinical trial discussed below and is currently being assessed in a Phase 1 trial in patients with chronic HIV-1 infection (NCT01734850). We are planning to assess the new sh143 target in combination with shPromA contained within the Cal-1 lentivirus construct backbone in the BLT model to determine whether a multiplexed approach can entrench enforced HIV-1 lockdown at the transcriptional level.

## Gene Therapy Approaches to HIV-1 Treatment

In 2009, the extraordinary success of utilizing bone marrow transplant to deliver HPSC from a CCR5-Δ32 homozygous donor opened the door for gene therapy approaches to HIV-1 treatment. The “Berlin patient” effectively received a functional cure for HIV-1, and currently 6 years post-transplantation, does not require ART and has no detectable pVL or proviral DNA (84, 85). Although this truly represents a modern day medical success, the circumstances of aggressive malignant disease and the rarity of matching HLA/CCR5-Δ32 homozygous donors present a formidable challenge in repeating this feat [reviewed in Ref. (50)] Further, this approach has been tried in six other patients without success (86). There are, however, several gene therapy clinical trials in various stages of completion involving HIV-1 infected patients with non-malignancies using constructs aimed at knocking down CCR5 to protect cells from HIV-1 infection. These current gene therapy clinical trials are summarized in Table 1.

**Table 1:** **Current HIV-1 gene therapy clinical trials**.

HIV-1 therapeutic target	Sponsor	Phase	Trial status	Reference
Autologous T cells genetically modified at the CCR5 gene by ZFN	Sangamo Biosciences	1/2	Completed	NCT01252641
WT-gag-TCR modified T cells or α/6-gag-TCR modified T cells	UPENN/Adaptimmune	1	Completed	NCT00991224
CD34^+^ HSPC transduced with two ribozyme sequences “L-TR/Tat-neo”	Ribozyome	2	Completed	NCT00002221
Autologous CD34^+^ HSPC transduced with anti-HIV-1 ribozyme (OZ1) targeting Tat/Vpr	Janssen-Cilag Pty Ltd.	2	Completed	NCT00074997 (52)
CD34^+^HSPC transduced with dual shRNAs targeting Tat/Rev and TAR decoy and CCR5 ribozyme	City of Hope Medical Center/NCI	Pilot	Active	NCT00569985 NCT01153646 (53)
Busulfan drug LVrHIV7-shI-TAR-CCR5RZ-transduced HSPC	City of Hope Medical Center/NCI	1	Recruiting	NCT01961063 (51, 54)
Autologous CD4^+^ T cells genetically modified at the CCR5 gene by ZFN SB-728mR	UPENN/NIAID	1	Recruiting	NCT02388594
Busulfan drug LVsh5/C46 (known as Cal-1) transduced HSPC or CD4^+^ T cells	Calimmune Inc.	1 2	Recruiting	NCT01734850 (85)
Long-term safety follow-up of Cal-1 recipients	Calimmune Inc.		Recruiting	NCT02390297

*ZFN, zinc finger nucleases; HSPC, hematopoetic stem progenitor cell; LV, lentiviral vector; NCI, National Cancer Institute; NIAID, National Institute of Allergy and Infectious Diseases; WT, wild-type; TCR, T cell receptor; NCT ID, National Clinical Trials Identifier; UPENN, University of Pennsylvania*.

*Accessed in https://clinicaltrials.gov April 2, 2015*.

One of the preclinical studies assessing safety and efficacy of an anti-HIV-1 lentiviral vector containing CCR5 shRNA and the C46 fusion inhibitor, termed Cal-1, has recently been reported (87). Effective delivery of LVsh5/C46 (Cal-1) was demonstrated in human T cell lines, PBMCs, CD4^+^ T cells, and CD34^+^ HSPCs, with both the CCR5-shRNA and C46 peptide being stably expressed in the target cells (87). Importantly, the study showed the Cal-1 construct was able to effectively protect gene-modified cells from both CXCR4- and CCR5-tropic HIV-1 strains (87). Further, the Cal-1 construct treatment was shown to be non-toxic, non-inflammatory, and had no adverse effect on HSPC differentiation (87). These encouraging data have led to the Cal-1 construct currently being tested in a Phase 1/2 clinical trial for the treatment of HIV-1 (Table 1).

Our envisaged future application of the TGS-inducing PromA and 143 sequences as a proposed therapeutic strategy is outlined in Figure 1C. The approach would be to use shPromA/sh143 TGS-inducing constructs to enforce latency in HIV-1 positive individuals who have cART-suppressed pVL and latent viral reservoirs. This strategy may provide a functional cure, by inducing and enforcing HIV-1 latency, thereby maintaining transcriptionally inactive virus and effectively render patients free from cART.

## Conclusion

In terms of approaches to a functional cure for HIV-1, ncRNA-mediated transcriptional regulation of HIV-1, particularly in the context of the viral reservoir, is starkly juxtaposed to current activation approaches, which rely on pan T-cell activation or extensive histone acetylation modification and are associated with substantial off-target responses. Instead, the ncRNA approach relies on sequence specificity to provide a highly focused approach in manipulation of the latent reservoir. Developing ncRNA-therapeutic approaches to control HIV-1 may have the potential to enforce HIV-1 latency and block initial infection, allowing control of the viral reservoir, free from traditional antiretroviral therapies.

## Author Contributions

CA, AK, KS, and GS wrote the manuscript; and KM prepared the figures.

## Conflict of Interest Statement

We declare there is no conflict of interest. Chantelle L. Ahlenstiel, Anthony D. Kelleher, and Kazuo Suzuki hold a patent for si/shRNA sequences targeting HIV-1. Geoff P. Symonds works for Calimmune Inc.

## Abbreviations

AP-1, activator protein 1; CTL, cytotoxic T lymphocyte; H3K27me3, histone 3 lysine 27 tri-methylation; H3K9me2, histone 3 lysine 9 di-methylation; HSPC, hematopoetic stem progenitor cell; LTR, long terminal repeat; LV, lentiviral vector; NF-κB, nuclear factor kappa B; NFAT, nuclear factor of activated T cells; SP1, specificity protein 1; TCR, T cell receptor; TGS, transcriptional gene silencing; WT, wild-type; ZFN, zinc finger nucleases.

## References

[1] Consortium EP Birney E Stamatoyannopoulos JA Dutta A Guigo R Gingeras TR Identification and analysis of functional elements in 1% of the human genome by the ENCODE pilot project. Nature (2007) 447:799–816. 10.1038/nature05874 17571346 PMC2212820

[2] Kaikkonen MU Lam MT Glass CK. Non-coding RNAs as regulators of gene expression and epigenetics. Cardiovasc Res (2011) 90:430–40. 10.1093/cvr/cvr097 21558279 PMC3096308

[3] Malecova B Morris KV. Transcriptional gene silencing through epigenetic changes mediated by non-coding RNAs. Curr Opin Mol Ther (2010) 12:214–22. 20373265 PMC2861437

[4] Morris KV Mattick JS. The rise of regulatory RNA. Nat Rev Genet (2014) 15:423–37. 10.1038/nrg3722 24776770 PMC4314111

[5] Amaral PP Mattick JS. Noncoding RNA in development. Mamm Genome (2008) 19:454–92. 10.1007/s00335-008-9136-7 18839252

[6] Stefani G Slack FJ. Small non-coding RNAs in animal development. Nat Rev Mol Cell Biol (2008) 9:219–30. 10.1038/nrm2347 18270516

[7] Gibb EA Brown CJ Lam WL. The functional role of long non-coding RNA in human carcinomas. Mol Cancer (2011) 10:38. 10.1186/1476-4598-10-38 21489289 PMC3098824

[8] Méndez C Ahlenstiel CL Kelleher AD. Post-transcriptional gene silencing, transcriptional gene silencing and HIV. World J Virol (2015) 4(3):219–44. 10.5501/wjv.v4.i3.219 26279984 PMC4534814

[9] Suzuki K Ahlenstiel C Marks K Kelleher AD. Promoter targeting RNAs: unexpected contributors to the control of HIV-1 transcription. Mol Ther Nucleic Acids (2015) 4:e222. 10.1038/mtna.2014.67 25625613 PMC4345301

[10] du Chene I Basyuk E Lin YL Triboulet R Knezevich A Chable-Bessia C Suv39H1 and HP1gamma are responsible for chromatin-mediated HIV-1 transcriptional silencing and post-integration latency. EMBO J (2007) 26:424–35. 10.1038/sj.emboj.7601517 17245432 PMC1783455

[11] Jordan A Bisgrove D Verdin E. HIV reproducibly establishes a latent infection after acute infection of T cells in vitro. EMBO J (2003) 22:1868–77. 10.1093/emboj/cdg188 12682019 PMC154479

[12] Van Lint C Emiliani S Ott M Verdin E. Transcriptional activation and chromatin remodeling of the HIV-1 promoter in response to histone acetylation. EMBO J (1996) 15:1112–20. 8605881 PMC450009

[13] Verdin E Paras P Jr Van Lint C. Chromatin disruption in the promoter of human immunodeficiency virus type 1 during transcriptional activation. EMBO J (1993) 12:3249–59. 8344262 10.1002/j.1460-2075.1993.tb05994.x PMC413592

[14] Williams SA Chen LF Kwon H Fenard D Bisgrove D Verdin E Prostratin antagonizes HIV latency by activating NF-kappaB. J Biol Chem (2004) 279:42008–17. 10.1074/jbc.M402124200 15284245

[15] Williams SA Chen LF Kwon H Ruiz-Jarabo CM Verdin E Greene WC. NF-kappaB p50 promotes HIV latency through HDAC recruitment and repression of transcriptional initiation. EMBO J (2006) 25:139–49. 10.1038/sj.emboj.7600900 16319923 PMC1356344

[16] Hsu DC Sereti I Ananworanich J. Serious non-AIDS events: immunopathogenesis and interventional strategies. AIDS Res Ther (2013) 10:29. 10.1186/1742-6405-10-29 24330529 PMC3874658

[17] Ho DD Neumann AU Perelson AS Chen W Leonard JM Markowitz M. Rapid turnover of plasma virions and CD4 lymphocytes in HIV-1 infection. Nature (1995) 373:123–6. 10.1038/373123a0 7816094

[18] Dinoso JB Kim SY Wiegand AM Palmer SE Gange SJ Cranmer L Treatment intensification does not reduce residual HIV-1 viremia in patients on highly active antiretroviral therapy. Proc Natl Acad Sci U S A (2009) 106:9403–8. 10.1073/pnas.0903107106 19470482 PMC2685743

[19] Wong JK Hezareh M Gunthard HF Havlir DV Ignacio CC Spina CA Recovery of replication-competent HIV despite prolonged suppression of plasma viremia. Science (1997) 278:1291–5. 10.1126/science.278.5341.1291 9360926

[20] Chun TW Justement JS Moir S Hallahan CW Maenza J Mullins JI Decay of the HIV reservoir in patients receiving antiretroviral therapy for extended periods: implications for eradication of virus. J Infect Dis (2007) 195:1762–4. 10.1086/518250 17492591

[21] Mellors JW Rinaldo CR Jr Gupta P White RM Todd JA Kingsley LA. Prognosis in HIV-1 infection predicted by the quantity of virus in plasma. Science (1996) 272:1167–70. 10.1126/science.272.5265.1167 8638160

[22] Perelson AS Neumann AU Markowitz M Leonard JM Ho DD. HIV-1 dynamics in vivo: virion clearance rate, infected cell life-span, and viral generation time. Science (1996) 271:1582–6. 10.1126/science.271.5255.1582 8599114

[23] Gulick RM Mellors JW Havlir D Eron JJ Gonzalez C McMahon D Treatment with indinavir, zidovudine, and lamivudine in adults with human immunodeficiency virus infection and prior antiretroviral therapy. N Engl J Med (1997) 337:734–9. 10.1056/NEJM199709113371102 9287228

[24] Palella FJ Jr Delaney KM Moorman AC Loveless MO Fuhrer J Satten GA Declining morbidity and mortality among patients with advanced human immunodeficiency virus infection. HIV Outpatient Study Investigators. N Engl J Med (1998) 338:853–60. 9516219 10.1056/NEJM199803263381301

[25] Koelsch KK Boesecke C McBride K Gelgor L Fahey P Natarajan V Impact of treatment with raltegravir during primary or chronic HIV infection on RNA decay characteristics and the HIV viral reservoir. AIDS (2011) 25:2069–78. 10.1097/QAD.0b013e32834b9658 21860347

[26] Yukl SA Shergill AK McQuaid K Gianella S Lampiris H Hare CB Effect of raltegravir-containing intensification on HIV burden and T-cell activation in multiple gut sites of HIV-positive adults on suppressive antiretroviral therapy. AIDS (2010) 24:2451–60. 10.1097/QAD.0b013e32833ef7bb 20827162 PMC2997807

[27] McMahon D Jones J Wiegand A Gange SJ Kearney M Palmer S Short-course raltegravir intensification does not reduce persistent low-level viremia in patients with HIV-1 suppression during receipt of combination antiretroviral therapy. Clin Infect Dis (2010) 50:912–9. 10.1086/650749 20156060 PMC2897152

[28] Hammer SM Ribaudo H Bassett R Mellors JW Demeter LM Coombs RW A randomized, placebo-controlled trial of abacavir intensification in HIV-1-infected adults with virologic suppression on a protease inhibitor-containing regimen. HIV Clin Trials (2010) 11:312–24. 10.1310/hct1106-312 21239359 PMC3108099

[29] Gandhi RT Zheng L Bosch RJ Chan ES Margolis DM Read S The effect of raltegravir intensification on low-level residual viremia in HIV-infected patients on antiretroviral therapy: a randomized controlled trial. PLoS Med (2010) 7:e1000321. 10.1371/journal.pmed.1000321 20711481 PMC2919424

[30] Gandhi RT Bosch RJ Aga E Albrecht M Demeter LM Dykes C No evidence for decay of the latent reservoir in HIV-1-infected patients receiving intensive enfuvirtide-containing antiretroviral therapy. J Infect Dis (2010) 201:293–6. 10.1086/649569 20001856 PMC2887684

[31] Archin NM Cheema M Parker D Wiegand A Bosch RJ Coffin JM Antiretroviral intensification and valproic acid lack sustained effect on residual HIV-1 viremia or resting CD4+ cell infection. PLoS One (2010) 5:e9390. 10.1371/journal.pone.0009390 20186346 PMC2826423

[32] Archin NM Liberty AL Kashuba AD Choudhary SK Kuruc JD Crooks AM Administration of vorinostat disrupts HIV-1 latency in patients on antiretroviral therapy. Nature (2012) 487:482–5. 10.1038/nature11286 22837004 PMC3704185

[33] Contreras X Schweneker M Chen CS McCune JM Deeks SG Martin J Suberoylanilide hydroxamic acid reactivates HIV from latently infected cells. J Biol Chem (2009) 284:6782–9. 10.1074/jbc.M807898200 19136668 PMC2652322

[34] Wang FX Xu Y Sullivan J Souder E Argyris EG Acheampong EA IL-7 is a potent and proviral strain-specific inducer of latent HIV-1 cellular reservoirs of infected individuals on virally suppressive HAART. J Clin Invest (2005) 115:128–37. 10.1172/JCI200522574 15630452 PMC539197

[35] Wightman F Ellenberg P Churchill M Lewin SR. HDAC inhibitors in HIV. Immunol Cell Biol (2012) 90:47–54. 10.1038/icb.2011.95 22083528

[36] Davey RT Jr Bhat N Yoder C Chun TW Metcalf JA Dewar R HIV-1 and T cell dynamics after interruption of highly active antiretroviral therapy (HAART) in patients with a history of sustained viral suppression. Proc Natl Acad Sci U S A (1999) 96:15109–14. 10.1073/pnas.96.26.15109 10611346 PMC24781

[37] van Praag RM Prins JM Roos MT Schellekens PT Ten Berge IJ Yong SL OKT3 and IL-2 treatment for purging of the latent HIV-1 reservoir in vivo results in selective long-lasting CD4+ T cell depletion. J Clin Immunol (2001) 21:218–26. 10.1023/A:1011091300321 11403229

[38] Siliciano RF Greene WC HIV latency. Cold Spring Harb Perspect Med (2011) 1:a007096 10.1101/cshperspect.a007096 22229121 PMC3234450

[39] Paiardini M Editorial: hijacking the IL-7/IL-7R system in HIV infection. J Leukoc Biol (2011) 89:491–3. 10.1189/jlb.1110614 21454359

[40] McKernan LN Momjian D Kulkosky J. Protein kinase C: one pathway towards the eradication of latent HIV-1 reservoirs. Adv Virol (2012) 2012:805347. 10.1155/2012/805347 22500169 PMC3303757

[41] Weissman D Dybul M Daucher MB Davey RT Jr Walker RE Kovacs JA. Interleukin-2 up-regulates expression of the human immunodeficiency virus fusion coreceptor CCR5 by CD4+ lymphocytes in vivo. J Infect Dis (2000) 181:933–8. 10.1086/315303 10720515

[42] Sanchez-Duffhues G Vo MQ Perez M Calzado MA Moreno S Appendino G Activation of latent HIV-1 expression by protein kinase C agonists. A novel therapeutic approach to eradicate HIV-1 reservoirs. Curr Drug Targets (2011) 12:348–56. 10.2174/138945011794815266 20955147

[43] Duvic M Talpur R Ni X Zhang C Hazarika P Kelly C Phase 2 trial of oral vorinostat (suberoylanilide hydroxamic acid, SAHA) for refractory cutaneous T-cell lymphoma (CTCL). Blood (2007) 109:31–9. 10.1182/blood-2006-06-025999 16960145 PMC1785068

[44] Edelstein LC Micheva-Viteva S Phelan BD Dougherty JP. Short communication: activation of latent HIV type 1 gene expression by suberoylanilide hydroxamic acid (SAHA), an HDAC inhibitor approved for use to treat cutaneous T cell lymphoma. AIDS Res Hum Retroviruses (2009) 25:883–7. 10.1089/aid.2008.0294 19689202 PMC2828260

[45] Heider U Rademacher J Lamottke B Mieth M Moebs M von Metzler I Synergistic interaction of the histone deacetylase inhibitor SAHA with the proteasome inhibitor bortezomib in cutaneous T cell lymphoma. Eur J Haematol (2009) 82:440–9. 10.1111/j.1600-0609.2009.01239.x 19220424

[46] Wei DG Chiang V Fyne E Balakrishnan M Barnes T Graupe M Histone deacetylase inhibitor romidepsin induces HIV expression in CD4 T cells from patients on suppressive antiretroviral therapy at concentrations achieved by clinical dosing. PLoS Pathog (2014) 10:e1004071. 10.1371/journal.ppat.1004071 24722454 PMC3983056

[47] Battistini A Sgarbanti M. HIV-1 latency: an update of molecular mechanisms and therapeutic strategies. Viruses (2014) 6:1715–58. 10.3390/v6041715 24736215 PMC4014718

[48] Ho YC Shan L Hosmane NN Wang J Laskey SB Rosenbloom DI Replication-competent noninduced proviruses in the latent reservoir increase barrier to HIV-1 cure. Cell (2013) 155:540–51. 10.1016/j.cell.2013.09.020 24243014 PMC3896327

[49] Jones RB O’Connor R Mueller S Foley M Szeto GL Karel D Histone deacetylase inhibitors impair the elimination of HIV-infected cells by cytotoxic T-lymphocytes. PLoS Pathog (2014) 10:e1004287. 10.1371/journal.ppat.1004287 25122219 PMC4133386

[50] Burke BP Boyd MP Impey H Breton LR Bartlett JS Symonds GP CCR5 as a natural and modulated target for inhibition of HIV. Viruses (2014) 6:54–68. 10.3390/v6010054 24381033 PMC3917431

[51] DiGiusto DL Krishnan A Li L Li H Li S Rao A RNA-based gene therapy for HIV with lentiviral vector-modified CD34(+) cells in patients undergoing transplantation for AIDS-related lymphoma. Sci Transl Med (2010) 2:36ra43. 10.1126/scitranslmed.3000931 20555022 PMC3130552

[52] ter Brake O t Hooft K Liu YP Centlivre M von Eije KJ Berkhout B. Lentiviral vector design for multiple shRNA expression and durable HIV-1 inhibition. Mol Ther (2008) 16:557–64. 10.1038/sj.mt.6300382 28178502

[53] Mitsuyasu RT Merigan TC Carr A Zack JA Winters MA Workman C Phase 2 gene therapy trial of an anti-HIV ribozyme in autologous CD34+ cells. Nat Med (2009) 15:285–92. 10.1038/nm.1932 19219022 PMC2768566

[54] Centlivre M Legrand N Klamer S Liu YP Jasmijn von Eije K Bohne M Preclinical in vivo evaluation of the safety of a multi-shRNA-based gene therapy against HIV-1. Mol Ther Nucleic Acids (2013) 2:e120. 10.1038/mtna.2013.48 24002730 PMC3808742

[55] Matzke MA Primig M Trnovsky J Matzke AJ. Reversible methylation and inactivation of marker genes in sequentially transformed tobacco plants. EMBO J (1989) 8:643–9. 16453872 10.1002/j.1460-2075.1989.tb03421.x PMC400855

[56] Wassenegger M Heimes S Riedel L Sanger HL. RNA-directed de novo methylation of genomic sequences in plants. Cell (1994) 76:567–76. 10.1016/0092-8674(94)90119-8 8313476

[57] Mette MF Aufsatz W van der Winden J Matzke MA Matzke AJ. Transcriptional silencing and promoter methylation triggered by double-stranded RNA. EMBO J (2000) 19:5194–201. 10.1093/emboj/19.19.5194 11013221 PMC302106

[58] Lippman Z May B Yordan C Singer T Martienssen R. Distinct mechanisms determine transposon inheritance and methylation via small interfering RNA and histone modification. PLoS Biol (2003) 1:E67. 10.1371/journal.pbio.0000067 14691539 PMC300680

[59] Morris KV Chan SW Jacobsen SE Looney DJ. Small interfering RNA-induced transcriptional gene silencing in human cells. Science (2004) 305:1289–92. 10.1126/science.1101372 15297624

[60] Castanotto D Tommasi S Li M Li H Yanow S Pfeifer GP Short hairpin RNA-directed cytosine (CpG) methylation of the RASSF1A gene promoter in HeLa cells. Mol Ther (2005) 12:179–83. 10.1016/j.ymthe.2005.03.003 15963934

[61] Hawkins PG Santoso S Adams C Anest V Morris KV. Promoter targeted small RNAs induce long-term transcriptional gene silencing in human cells. Nucleic Acids Res (2009) 37:2984–95. 10.1093/nar/gkp127 19304753 PMC2685082

[62] Janowski BA Huffman KE Schwartz JC Ram R Nordsell R Shames DS Involvement of AGO1 and AGO2 in mammalian transcriptional silencing. Nat Struct Mol Biol (2006) 13:787–92. 10.1038/nsmb1140 16936728

[63] Kim DH Saetrom P Snove O Jr Rossi JJ. MicroRNA-directed transcriptional gene silencing in mammalian cells. Proc Natl Acad Sci U S A (2008) 105:16230–5. 10.1073/pnas.0808830105 18852463 PMC2571020

[64] Suzuki K Juelich T Lim H Ishida T Watanebe T Cooper DA Closed chromatin architecture is induced by an RNA duplex targeting the HIV-1 promoter region. J Biol Chem (2008) 283:23353–63. 10.1074/jbc.M709651200 18519571 PMC2516975

[65] Ting AH Schuebel KE Herman JG Baylin SB. Short double-stranded RNA induces transcriptional gene silencing in human cancer cells in the absence of DNA methylation. Nat Genet (2005) 37:906–10. 10.1038/ng1611 16025112 PMC2659476

[66] Weinberg MS Villeneuve LM Ehsani A Amarzguioui M Aagaard L Chen ZX The antisense strand of small interfering RNAs directs histone methylation and transcriptional gene silencing in human cells. RNA (2006) 12:256–62. 10.1261/rna.2235106 16373483 PMC1370905

[67] Suzuki K Shijuuku T Fukamachi T Zaunders J Guillemin G Cooper D Prolonged transcriptional silencing and CpG methylation induced by siRNAs targeted to the HIV-1 promoter region. J RNAi Gene Silencing (2005) 1:66–78. 19771207 PMC2737205

[68] Weinberg MS Barichievy S Schaffer L Han J Morris KV. An RNA targeted to the HIV-1 LTR promoter modulates indiscriminate off-target gene activation. Nucleic Acids Res (2007) 35:7303–12. 10.1093/nar/gkm847 17959645 PMC2175361

[69] Singh A Palanichamy JK Ramalingam P Kassab MA Bhagat M Andrabi R Long-term suppression of HIV-1C virus production in human peripheral blood mononuclear cells by LTR heterochromatization with a short double-stranded RNA. J Antimicrob Chemother (2014) 69:404–15. 10.1093/jac/dkt348 24022068

[70] Ahlenstiel CL Lim HG Cooper DA Ishida T Kelleher AD Suzuki K. Direct evidence of nuclear Argonaute distribution during transcriptional silencing links the actin cytoskeleton to nuclear RNAi machinery in human cells. Nucleic Acids Res (2012) 40:1579–95. 10.1093/nar/gkr891 22064859 PMC3287199

[71] Suzuki K Ishida T Yamagishi M Ahlenstiel C Swaminathan S Marks K Transcriptional gene silencing of HIV-1 through promoter targeted RNA is highly specific. RNA Biol (2011) 8:1035–46. 10.4161/rna.8.6.16264 21955498 PMC3256422

[72] Yamagishi M Ishida T Miyake A Cooper DA Kelleher AD Suzuki K Retroviral delivery of promoter-targeted shRNA induces long-term silencing of HIV-1 transcription. Microbes Infect (2009) 11:500–8. 10.1016/j.micinf.2009.02.003 19233310

[73] Suzuki K Hattori S Marks K Ahlenstiel C Maeda Y Ishida T Promoter targeting shRNA suppresses HIV-1 infection in vivo through transcriptional gene silencing. Mol Ther Nucleic Acids (2013) 2:e137. 10.1038/mtna.2013.64 24301868 PMC3894581

[74] Lim HG Suzuki K Cooper DA Kelleher AD. Promoter-targeted siRNAs induce gene silencing of simian immunodeficiency virus (SIV) infection in vitro. Mol Ther (2008) 16:565–70. 10.1038/sj.mt.6300380 18227841

[75] Ahlenstiel C Mendez C Lim STH Marks K Turville SG Cooper DA Novel RNA duplex locks HIV-1 in a latent state via chromatin-mediated transcriptional silencing. Mol Ther Nucleic Acids (2015). 10.1038/mtna.2015.31 PMC4881759 26506039

[76] Malim MH Bieniasz PDHIV. Restriction factors and mechanisms of evasion. Cold Spring Harb Perspect Med (2012) 2:a006940. 10.1101/cshperspect.a006940 22553496 PMC3331687

[77] Brummelkamp TR Bernards R Agami R. A system for stable expression of short interfering RNAs in mammalian cells. Science (2002) 296:550–3. 10.1126/science.1068999 11910072

[78] Okada S Harada H Ito T Saito T Suzu S. Early development of human hematopoietic and acquired immune systems in new born NOD/Scid/Jak3null mice intrahepatic engrafted with cord blood-derived CD34 + cells. Int J Hematol (2008) 88:476–82. 10.1007/s12185-008-0215-z 19039627

[79] Hattori S Ide K Nakata H Harada H Suzu S Ashida N Potent activity of a nucleoside reverse transcriptase inhibitor, 4’-ethynyl-2-fluoro-2’-deoxyadenosine, against human immunodeficiency virus type 1 infection in a model using human peripheral blood mononuclear cell-transplanted NOD/SCID Janus kinase 3 knockout mice. Antimicrob Agents Chemother (2009) 53:3887–93. 10.1128/AAC.00270-09 19546363 PMC2737856

[80] Shimizu S Hong P Arumugam B Pokomo L Boyer J Koizumi N A highly efficient short hairpin RNA potently down-regulates CCR5 expression in systemic lymphoid organs in the hu-BLT mouse model. Blood (2010) 115:1534–44. 10.1182/blood-2009-04-215855 20018916 PMC2830759

[81] Shimizu S Ringpis GE Marsden MD Cortado RV Wilhalme HM Elashoff D RNAi-mediated CCR5 knockdown provides HIV-1 resistance to memory T cells in humanized BLT mice. Mol Ther Nucleic Acids (2015) 4:e227. 10.1038/mtna.2015.3 25689223 PMC4345313

[82] An DS Donahue RE Kamata M Poon B Metzger M Mao SH Stable reduction of CCR5 by RNAi through hematopoietic stem cell transplant in non-human primates. Proc Natl Acad Sci U S A (2007) 104:13110–5. 10.1073/pnas.0705474104 17670939 PMC1941789

[83] van Lunzen J Glaunsinger T Stahmer I von Baehr V Baum C Schilz A Transfer of autologous gene-modified T cells in HIV-infected patients with advanced immunodeficiency and drug-resistant virus. Mol Ther (2007) 15:1024–33. 10.1038/mt.sj.6300124 17356541

[84] Hutter G Nowak D Mossner M Ganepola S Mussig A Allers K Long-term control of HIV by CCR5 Delta32/Delta32 stem-cell transplantation. N Engl J Med (2009) 360:692–8. 10.1056/NEJMoa0802905 19213682

[85] Allers K Hutter G Hofmann J Loddenkemper C Rieger K Thiel E Evidence for the cure of HIV infection by CCR5Delta32/Delta32 stem cell transplantation. Blood (2011) 117:2791–9. 10.1182/blood-2010-09-309591 21148083

[86] Verheyen J Esser S Kordelas L More on shift of HIV tropism in stem-cell transplantation with CCR5 delta32/delta32 mutation. N Engl J Med (2014) 371:2438 10.1056/NEJMc1412279 25517720

[87] Wolstein O Boyd M Millington M Impey H Boyer J Howe A Preclinical safety and efficacy of an anti–HIV-1 lentiviral vector containing a short hairpin RNA to CCR5 and the C46 fusion inhibitor. Mol Ther Methods Clin Dev (2014) 1:11. 10.1038/mtm.2013.11 26015947 PMC4365823

